# ATR and Chk1 Suppress a Caspase-3–Dependent Apoptotic Response Following DNA Replication Stress

**DOI:** 10.1371/journal.pgen.1000324

**Published:** 2009-01-02

**Authors:** Katie Myers, Mary E. Gagou, Pedro Zuazua-Villar, Rene Rodriguez, Mark Meuth

**Affiliations:** 1 Institute for Cancer Studies, School of Medicine and Biomedical Sciences, University of Sheffield, Sheffield, United Kingdom; National Institute of Diabetes and Digestive and Kidney Diseases, United States of America

## Abstract

The related PIK-like kinases Ataxia-Telangiectasia Mutated (ATM) and ATM- and Rad3-related (ATR) play major roles in the regulation of cellular responses to DNA damage or replication stress. The pro-apoptotic role of ATM and p53 in response to ionizing radiation (IR) has been widely investigated. Much less is known about the control of apoptosis following DNA replication stress. Recent work indicates that Chk1, the downstream phosphorylation target of ATR, protects cells from apoptosis induced by DNA replication inhibitors as well as IR. The aim of the work reported here was to determine the roles of ATM- and ATR-protein kinase cascades in the control of apoptosis following replication stress and the relationship between Chk1-suppressed apoptotic pathways responding to replication stress or IR. ATM and ATR/Chk1 signalling pathways were manipulated using siRNA-mediated depletions or specific inhibitors in two tumour cell lines or fibroblasts derived from patients with inherited mutations. We show that depletion of ATM or its downstream phosphorylation targets, NBS1 and BID, has relatively little effect on apoptosis induced by DNA replication inhibitors, while ATR or Chk1 depletion strongly enhances cell death induced by such agents in all cells tested. Furthermore, early events occurring after the disruption of DNA replication (accumulation of RPA foci and RPA34 hyperphosphorylation) in ATR- or Chk1-depleted cells committed to apoptosis are not detected in ATM-depleted cells. Unlike the Chk1-suppressed pathway responding to IR, the replication stress-triggered apoptotic pathway did not require ATM and is characterized by activation of caspase 3 in both p53-proficient and -deficient cells. Taken together, our results show that the ATR-Chk1 signalling pathway plays a major role in the regulation of death in response to DNA replication stress and that the Chk1-suppressed pathway protecting cells from replication stress is clearly distinguishable from that protecting cells from IR.

## Author Summary

The integrity of the genetic information in cells is protected by elaborate mechanisms that ensure that an accurate DNA copy is passed from generation to generation. These mechanisms repair errors in DNA sequence or stop growth if DNA structure is compromised. However, if the level of DNA damage is too severe, cells may also respond by inducing death rather than attempt repair. Relatively little is known about how cells decide whether to repair damage or commit to death. The purpose of our work was to identify genes that control this decision-making process while cells are duplicating DNA. We show that two genes play a major role in this process; however, our work also suggests considerable complexity in this death response as different death pathways are triggered in response to different forms of DNA damage. Since DNA replication inhibitors are used widely in the treatment of cancer, our work may enable us to more effectively kill cancer cells in treatment protocols employing these agents.

## Introduction

Cells respond to DNA damage by triggering cell cycle arrest, DNA repair, or death. The related PIK-like kinases ATM (Ataxia-Telangiectasia Mutated) and ATR (ATM- and Rad3-related) are major coordinators of this damage response [1]. ATM is central to the DNA double-strand break (DSB) response. It delays DNA synthesis and the onset of mitosis following DSB induction by agents such as ionizing radiation (IR) through a complex signalling cascade that includes p53, Chk2 and NBS1 as phosphorylation targets [2]–[4]. This signalling cascade also plays a major role in the onset of apoptosis following IR through the p53-mediated transcriptional activation of pro-apoptotic proteins such as BAX and PUMA [5]–[7]. However cells deficient in ATM are only partially defective in the induction of apoptosis by IR while p53 deficient cells show a more complete resistance [8],[9]. These observations indicate that both ATM-dependent and independent pathways regulate the induction of apoptosis by IR. Chk2 may be particularly important for the ATM-independent pathway as mouse cells with knockouts of both Chk2 and ATM show levels of apoptosis similar to those found in p53−/− cells [9].

ATR and its downstream phosphorylation target, Chk1, are generally activated in response to UV and agents that stall DNA replication forks [10],[11]. Activated Chk1 coordinates many of the cellular responses to replication fork stress. More specifically, it prevents the inappropriate firing of late replication origins, the abandonment of replication forks, and premature chromosome condensation following disruption of replication [12]–[15]. In contrast to the proapoptotic role of the ATM-mediated protein kinase cascade in the response to IR, Chk1 has an anti-apoptotic effect in the cellular response to replication inhibitors [13], [16]–[18] as well as IR [19]. SiRNA mediated ablation of Chk1 (but not Chk2) causes cells arrested in S-phase by a range of replication inhibitors to undergo apoptosis. This death response is p53 independent, but cells that lack both Chk1 and p21 show a more robust death response and reduced cell survival [17]. Thus the Chk1 pathway plays a key role in protecting S-phase cells from apoptosis during replication stress and p21 mediates this role, presumably by preventing entry into S-phase. Intriguingly depletion of the replication helicase cofactor Cdc45 that plays an essential role in DNA replication origin firing and fork elongation protects cells lacking Chk1 from undergoing apoptosis, suggesting that the role of Chk1 in controlling origin firing and maintaining fork integrity is key to its anti-apoptotic effect [20].

A role for Chk1 in the suppression of apoptosis in response to IR was revealed in a zebrafish embryo-based screen [19]. The novel death pathway triggered in p53 mutant embryos in the absence of Chk1 required ATM, ATR and caspase 2 but not other caspases. It was further shown that this response was not limited to Zebrafish as IR triggered a caspase-2 dependent apoptotic response in cultured p53-deficient human tumour cells treated with a Chk1 inhibitor. Taken together these reports establish a major role for Chk1 in the protection of cells from apoptosis in response to a wide range of DNA damage.

While the role of the ATM signalling cascade in the induction of apoptosis following IR is well established, relatively little is known concerning the contribution of this signalling pathway in response to replication fork stress. DNA replication inhibitors trigger a rapid ATM autophosphorylation and ATM-dependent phosphorylations of Chk2, NBS1 and p53 [21]. More recent work has implicated BID as a downstream ATM phosphorylation target and a suppressor of apoptosis in response to DNA replication inhibitors [22]. In the work reported here we compared the effects of ATM, NBS1 or BID deficiency on apoptosis with those obtained following siRNA-mediated depletion of ATR or Chk1. We show that depletion or loss of ATM, NBS1 or BID has little or no significant effect on the induction of apoptosis in two human tumour cell lines or immortalized human fibroblasts treated with DNA replication inhibitors, even though depletion of ATR or Chk1 in these cells led to high levels of cell death. Furthermore, unlike the Chk1 suppressed pathway responding to IR identified in Zebrafish embryos, the pathway regulated by Chk1 in response to replication inhibitors was triggered in both p53 proficient and deficient cell lines, did not require ATM or ATR, and was primarily characterized by caspase-3 activation.

## Results

### Effects of ATM, ATR, or Chk1 Depletion on the Induction of Apoptosis Following Replication Stress

**Figure 1:**
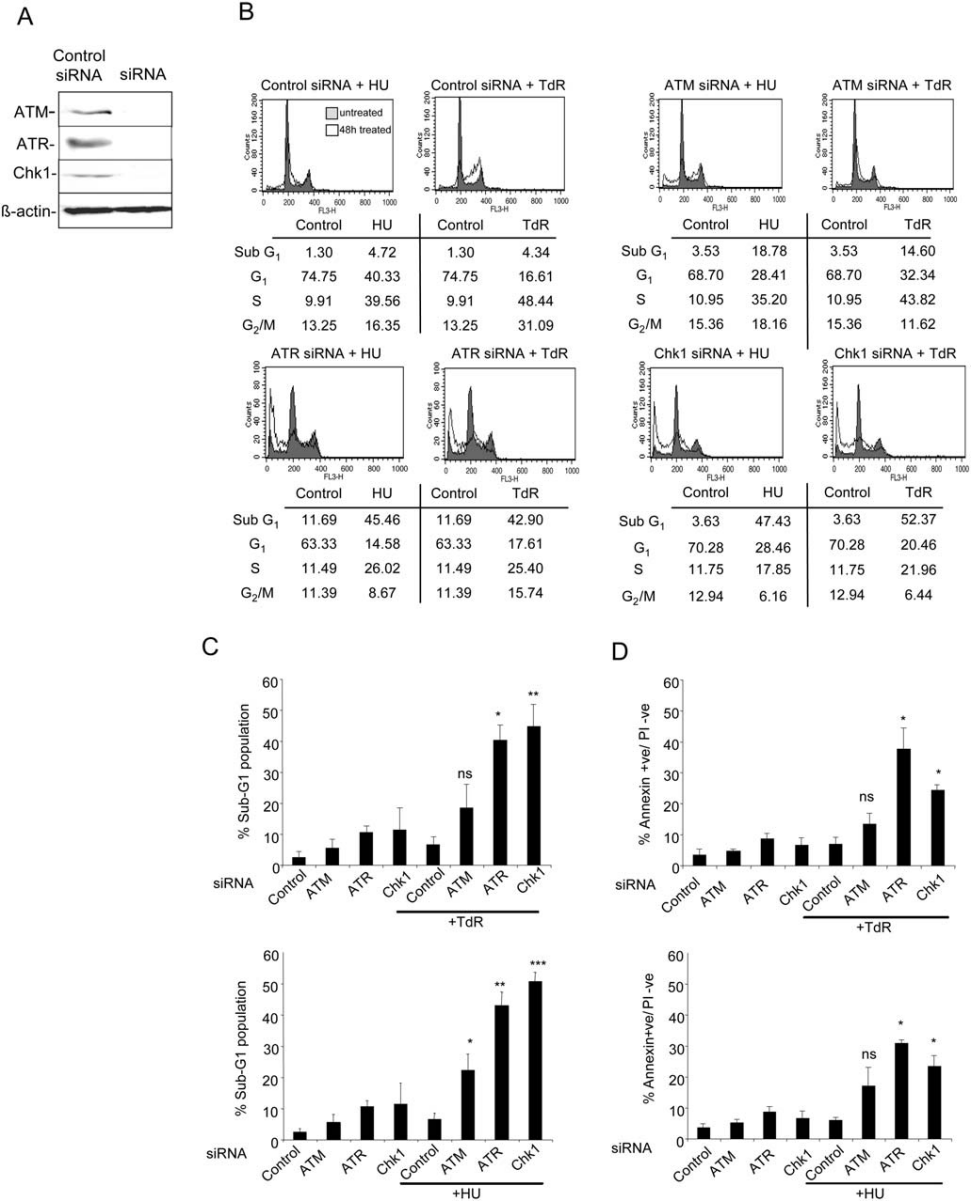
Effects of ATM, ATR or Chk1 depletion on the induction of apoptosis following replication fork stress. A) Protein levels 48 hours after transfection of the indicated siRNAs in HCT116 cells. β-actin levels are presented as a loading control. B) Representative cell cycle distributions of HCT116 cultures transfected with indicated siRNAs 24 hours before treatment with 2 mM thymidine or 0.5 mM hydroxyurea for 48 hours. Shaded profiles represent control cultures not treated with replication inhibitors, unshaded profiles represent cultures treated with HU or thymidine (TdR). C & D) Cultures of HCT116 cells transfected with the indicated siRNAs were treated 2 mM thymidine or 0.5 mM HU for 48 hours or left untreated as controls. Cells were then harvested and the level of apoptosis was determined by measuring the percentage of cells containing a subG1 DNA content by flow cytometry (C) or the percentage of the population binding Annexin V but not PI (D). Results in C and D represent the means of two to three independent experiments±standard deviations. Statistical significance versus thymidine or HU treated control siRNA transfected cells: ns, not significant; *p<.05; **p<.005; ***p<.005.

To determine the role of ATM or ATR relative to Chk1 in the regulation of apoptosis in response to DNA replication stress, HCT116 cells were treated with control, ATM, ATR, or Chk1 siRNAs (Figure 1A) for 24 hours before treatment with thymidine or HU. After 24 or 48 hour treatment with replication inhibitors, cells were fixed, stained with PI, and analysed for DNA content by flow cytometry or assayed for Annexin V binding. Cells treated for 24 hours with thymidine or HU accumulated in S-phase, however there was no significant increase in the level of cells with a subG1 DNA content in cultures treated with any of the siRNAs.

After a 48 hour exposure to thymidine or HU, cells treated with the control siRNA continued to accumulate in S as well as G2 but there was only a small increase in cells with a subG1 DNA content relative to controls (Figure 1B & C). HCT116 cells depleted of Chk1 or ATM showed a cell cycle distribution similar to that found for cultures treated with the control siRNA while there was a small increase in the fraction of cells showing a subG1 DNA content in cultures depleted of ATR. When Chk1 or ATR depleted cells were treated with thymidine or HU, fewer S or G2 DNA phase cells were detected relative to the cultures treated with the control siRNA while a markedly higher fraction of cells (40 to 50%) with a subG1 DNA content was evident (Figure 1B & C). ATM depleted HCT116 cells showed a somewhat different response following treatment with thymidine or HU. The fraction of S phase cells increased in these cultures like cultures treated with control siRNA (Figure 1B). However after thymidine treatment, ATM-depleted cells arrested earlier in S-phase while HU treated cells showed a higher frequency of cells with a late S-phase DNA content. There was an increase in the level of cells with a subG1 DNA content relative to control cultures after a 48 hour exposure to the inhibitors. This reached significance for HU treated cells but not those treated with thymidine. Notably the fraction of subG1 cells was consistently lower in ATM depleted cells relative to Chk1 or ATR depleted cultures (Figure 1B & C). Similarly, there was little effect on the fraction of cells with a subG1 DNA content in HCT116 cells treated with the ATM inhibitor KU-55933 [23] following a 48 hour exposure to thymidine (Figure S1). p53 defective SW480 cells depleted of Chk1 or ATR also had a significantly higher level of subG1 cells than those depleted of ATM following a 48 hour treatment with HU (Figure S2).

Analysis of Annexin V+ cells gave similar results (Figure 1D). HCT116 cultures depleted of either Chk1 or ATR showed a significant increase in the level of Annexin V+ cells relative to those treated with the control siRNA following either thymidine or HU treatment. The fraction of Annexin V+ cells in ATM depleted cultures exposed to thymidine or HU increased relative to control siRNA treated cells but this did not reach significance for either replication inhibitor.

### Co-Depletion of ATM or ATR and Chk1 Does Not Further Enhance or Suppress Apoptosis in Response to Replication Inhibitors

**Figure 2:**
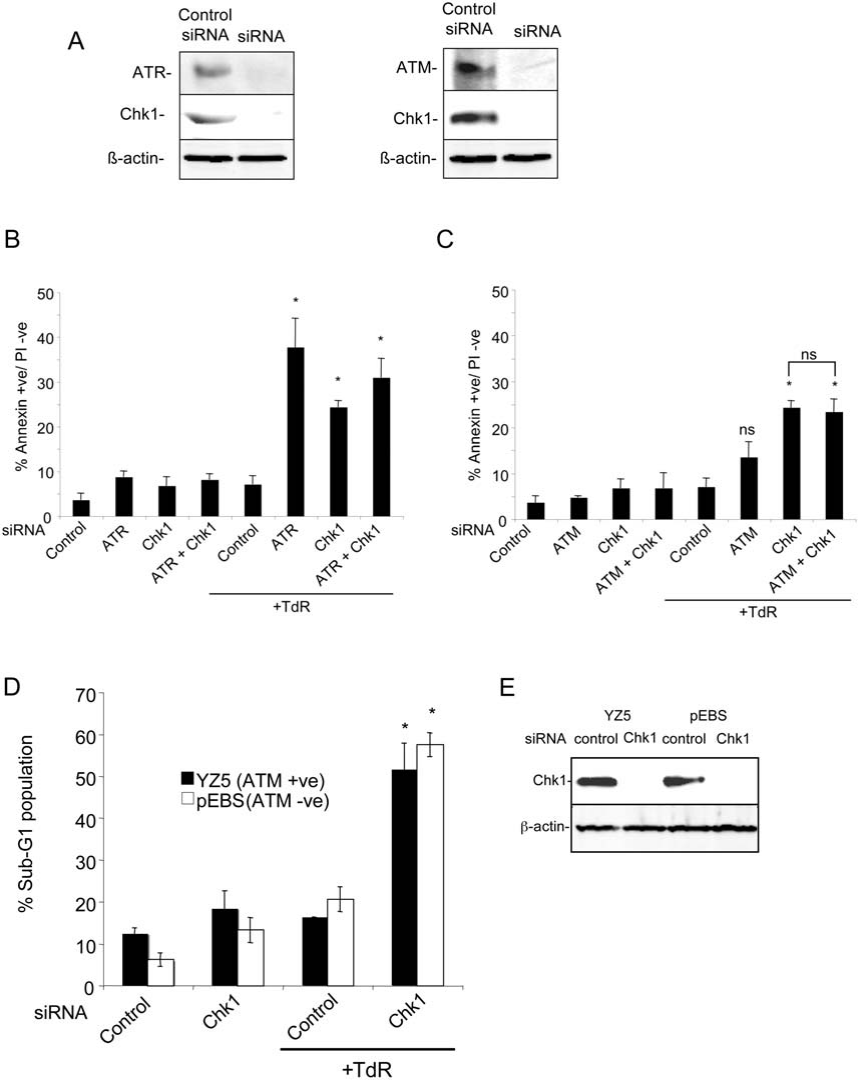
Effects of co-depletions of ATM, ATR or Chk1 on apoptosis in response to replication inhibitors. A) ATR and Chk1 protein levels 48 hours after transfection of the indicated combinations of siRNAs in HCT116 cells. β-actin levels are presented as loading controls. B & C) Level of apoptotic cells in HCT116 cultures (measured by Annexin V binding) after transfection of the indicated siRNAs following a 48 hour treatment with 2 mM thymidine. D) Induction of apoptosis (measured as the sub-G1 population) in ATM corrected YZ5 or ATM deficient pEBS cells transfected with control or Chk1 siRNAs and treated or not treated with 2 mM thymidine for 48 hours. E) Western blot analysis of Chk1 protein levels in the ATM-deficient pEBS or corrected YZ5 cells after 48 hours treatment with the relevant siRNA. β-actin levels are presented as loading controls. Results in B–D represent the means of two to three independent experiments±standard deviations. Statistical significance versus thymidine or HU treated control siRNA transfected cells: ns, not significant; *p<.05.

To determine whether the depletions of these checkpoint proteins affected apoptosis through related pathways, HCT116 cells were treated with combinations of siRNAs for the checkpoint proteins (Figure 2A). Apoptotic responses after treatment with thymidine were measured by the AnnexinV assay. In cultures depleted of both Chk1 and ATR, the increased level of AnnexinV+ cells was not significantly different from that produced by depletion of either protein alone (Figure 2B). When HCT116 cells were depleted of both Chk1 and ATM there was no significant difference in the fraction of AnnexinV+ cells relative to cultures depleted of Chk1 alone, although the level of apoptotic cells was significantly higher than that found in cells depleted of ATM alone (Figure 2C).

We further examined the induction of apoptosis following treatment with replication inhibitors in an immortalized human fibroblast line derived from an AT patient (pEBS) and a derivative of this line corrected for the ATM defect (YZ5) [24]. In these cultures, the level of pEBS fibroblasts with a subG1 DNA content was not significantly different from that found in the ATM corrected cells following treatment with thymidine (Figure 2D). However, cultures of both pEBS and YZ5 depleted of Chk1 (Figure 2E) showed a significantly higher level of cells with a subG1 DNA content relative to controls following thymidine treatment (Figure 2D). Furthermore there was no significant difference in fraction of cells with a subG1 DNA content in the two lines. Thus our results suggest that Chk1 and ATR regulate apoptosis in response to replication stress through a common pathway while ATM does not play a significant role in this response.

### Enhanced Levels of RPA Foci in Cells Depleted of Chk1 or ATR but not Those Depleted of ATM in Response to Replication Inhibitors

**Figure 3:**
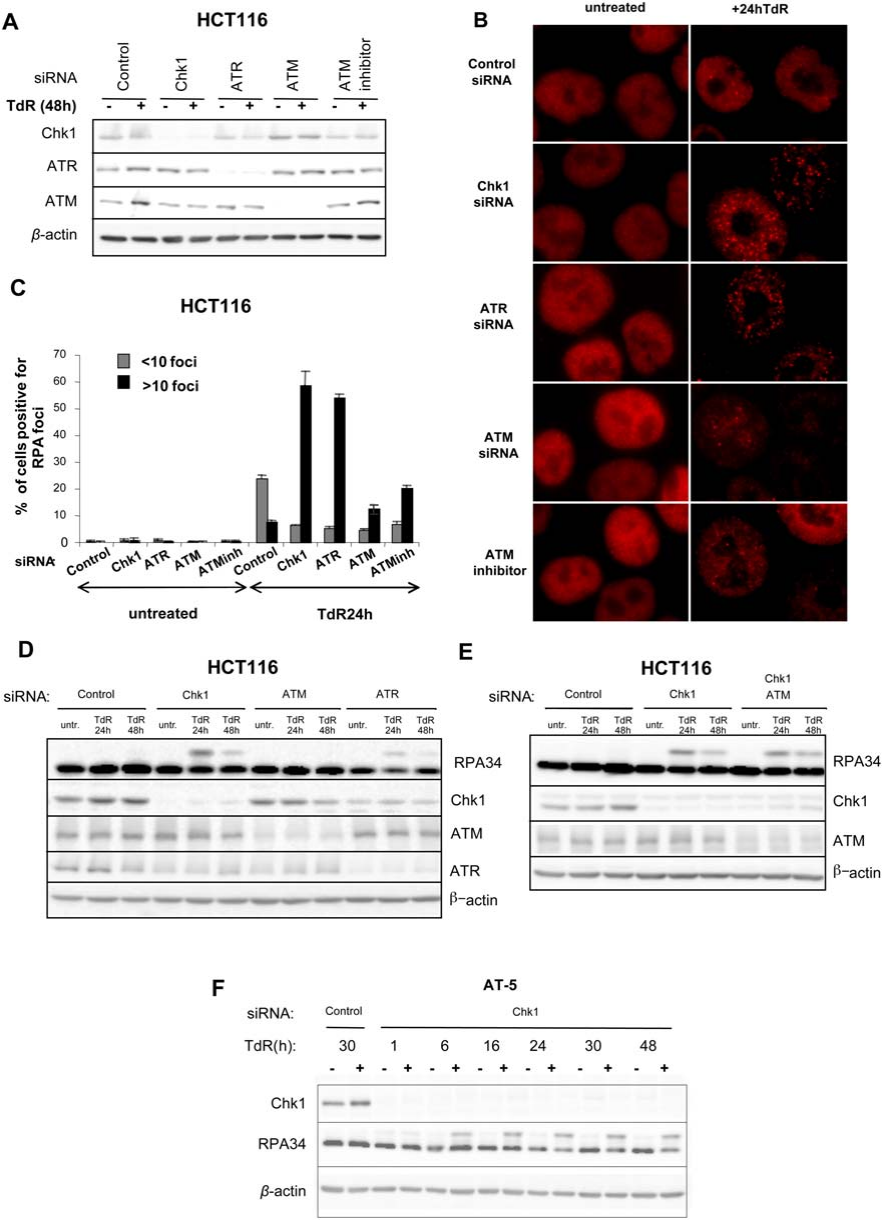
Induction of RPA foci and RPA34 hyperphosphorylation in cells depleted of Chk1 or ATR but not ATM deficient cells following thymidine treatment. A) Levels of Chk1, ATR, or ATM proteins following treatment of HCT116 cells with the indicated siRNAs or 10 µM of the ATM inhibitor (KU55399). B) Representative images of RPA foci obtained by immunostaining of HCT116 cells treated with control, Chk1, ATR, or ATM siRNAs or 10 µM of the ATM inhibitor KU55399 after a 24 hour thymidine treatment. C) Percentages of cells treated with the indicated siRNAs or 10 µM of the ATM inhibitor KU55399 presenting low (<10 foci/cell) or high (>10 foci/cell) levels of RPA foci after a 24 hour exposure to thymidine. D) Western blot analysis of RPA34, Chk1, ATR and ATM in extracts from HCT116 cells transfected with the indicated siRNAs and exposed to 2 mM thymidine for the indicated times. The band showing slower mobility on panel probed with the RPA34 antibody represents hyperphosphorylated forms of the protein. E) Hyperphosphorylation of RPA34 in HCT116 cells co-depleted of Chk1 and ATM after exposure to 2 mM thymidine for the indicated times. F) Hyperphosphorylation of RPA34 in the AT fibroblast line AT5 transfected with control or Chk1 siRNAs following exposure to 2 mM thymidine for the indicated times. Hyperphosphorylated RPA34 was detected by six hours after thymidine treatment. AT5 cells treated with the control siRNA do not show the slower mobility band characteristic of RPA34 hyperphosphorylation following a 30 hour thymidine treatment.

RPA foci appear as an early event in Chk1 depleted cells in response to replication inhibitors [20]. We next determined whether cells depleted of ATR or ATM showed induction of such foci. HCT116 cells were treated with control, Chk1, ATR, or ATM siRNAs for 24 hours before treatment with thymidine (Figure 3A). After 24 hours cells were fixed and stained for RPA. Following depletion of Chk1 or ATR, RPA foci accumulated (>10 foci/cell) in up to 50 to 60% of cells following treatment with thymidine (Figure 3B & C). In contrast cultures depleted of ATM or treated with the ATM inhibitor KU-55933 showed a significantly lower percentage of cells accumulating these foci. In addition hyperphosphorylation of RPA 34 was evident in Chk1 or ATR depleted HCT116 cells after thymidine treatment but not cells depleted of ATM (Figure 3D). Since ATM has been reported to contribute to the phosphorylation of RPA34 following DNA damage [25], this decrease in the level of phosphorylation could simply be due to a decrease in ATM kinase activity. However, RPA34 hyperphosphorylation was also evident in HCT116 cells depleted of both Chk1 and ATM (Figure 3E). Furthermore AT5 fibroblasts (derived from AT patients) depleted of Chk1 showed hyperphosphorylation of RPA34 as early as six hours after thymidine treatment (Figure 3F) demonstrating that cells are still capable of RPA34 phosphorylation when ATM function is compromised. Thus the early events detected in Chk1- or ATR-depleted cells treated with replication inhibitors are not evident in ATM depleted cells.

### Caspase 3 Is Primarily Activated in Chk1-Depleted Cells but not Those Depleted of ATM Following Thymidine Treatment

**Figure 4:**
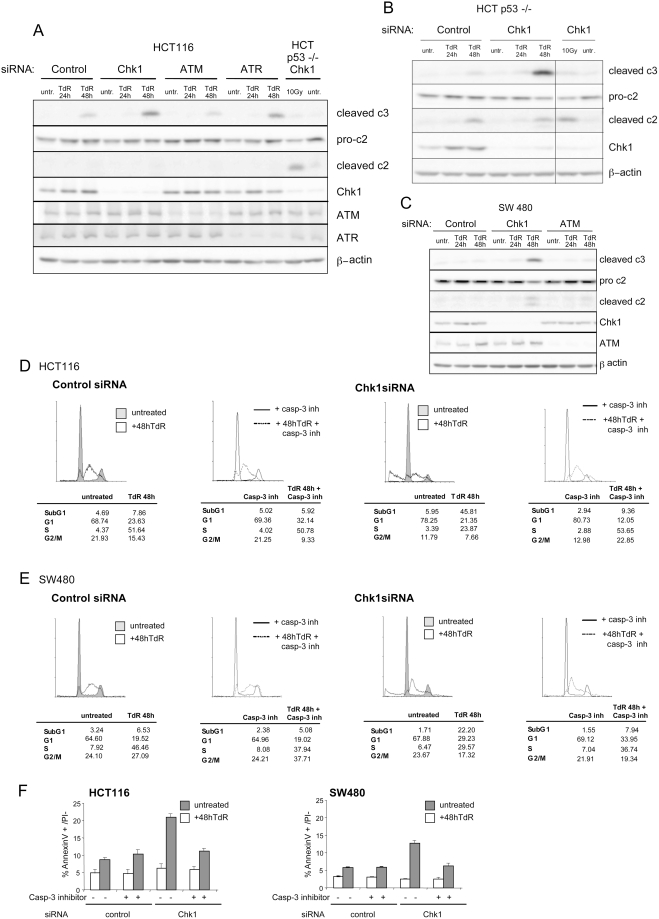
Induction of caspase 3 cleavage in HCT116 cells depleted of Chk1 following thymidine treatment. A) Western blot analysis of caspase 2 (c2) and cleaved caspase 3 (c3) in extracts obtained from HCT116 cells transfected with the indicated siRNAs treated or not treated with 2 mM thymidine for the indicated times. The two lanes furthest to the right present caspase 2 and cleaved caspase 3 levels in HCT116 p53−/− cells depleted of Chk1 exposed or not exposed to 10 Gy IR. The levels of Chk1, ATR and ATM in the cells treated with the indicated siRNAs are presented. β-actin levels are presented as loading controls. B) Western blot analysis of caspase 2 and cleaved caspase 3 in extracts obtained from HCT116 p53−/− cells transfected with control or Chk1 siRNAs and treated or not treated with 2 mM thymidine for the indicated times. As above, the two lanes furthest to the right present caspase 2 and cleaved caspase 3 levels in HCT116 p53−/− cells depleted of Chk1 exposed or not exposed to 10 Gy IR. The levels of Chk1 in the cells treated with the indicated siRNAs are presented. C) Western blot analysis of cleaved caspase 2 and cleaved caspase 3 in extracts obtained from SW480 cells transfected with control, Chk1, or ATM siRNAs and treated or not treated with 2 mM thymidine for the indicated times. D–E) Cell cycle analysis of HCT116 (D) or SW480 (E) cells transfected with the indicated siRNAs and treated or not treated with 2 mM thymdine for 48 hours. The indicated cultures were also treated with 100 mM of the caspase 3 inhibitor II, Z-DEVD-FMK. F) HCT116 and SW480 cultures treated as above were also assayed for AnnexinV+/PI negative cells.

We previously reported that caspase 3 was activated in HCT116 cells depleted of Chk1 following treatment with replication inhibitors [17]. To determine whether caspase 3 was activated in cells depleted of ATM, HCT116 cells treated with control, Chk1, ATR or ATM siRNAs were exposed to thymidine for 24 or 48 hours. Activated caspase 3 was assayed by Western blotting using cell free lysates prepared from these cultures. This analysis revealed a strong increase in the level of the activated (cleaved) form of caspase 3 in Chk1 or ATR depleted HCT116 cells exposed to thymidine (Figure 4A). A lower level of the activated caspase 3 was detected in HCT116 cells treated with control or ATM siRNAs following exposure to thymidine consistent with the lower level of apoptosis found in such cells.

Recently it was reported that p53 deficient cells treated with a Chk1 inhibitor or siRNA showed cleavage of caspase 2 but not caspase 3 following exposure to IR while p53+/+ HCT116 cells predominantly showed cleavage of caspase 3 [19]. To determine the effect of p53 status on caspase 2 and 3 cleavage in Chk1 depleted cells treated with thymidine, the cleaved forms of these caspases were analysed in HCT116 p53−/− cells treated with control or Chk1 siRNAs. In agreement with the previous report, cleaved caspase 2 was detected in Chk1 depleted HCT116 p53−/− cells exposed to 10 Gy IR while cleaved caspase 3 was not evident (Figure 4A & B). Following a 48 hour exposure of Chk1 depleted HCT116 p53−/− cells to thymidine, there was little change in the level of the cleaved caspase 2 relative to cells treated with the control siRNA while more robust levels of cleaved caspase 3 were evident (Figure 4B). Similarly Chk1 depleted SW480 cells (that are defective in p53 function) showed a weak increase in the level of cleaved caspase 2 while caspase 3 cleavage was clearly induced (Figure 4C). Cleaved caspase 2 was not detected in Chk1 depleted p53+/+ HCT116 cells following thymidine treatment (Figure 4A).

To determine whether the induction of apoptosis was dependent upon caspase 3 activation following thymidine exposure, Chk1 depleted HCT116 or SW480 cells exposed to thymidine were treated with the caspase 3 inhibitor II (Z-DEVD-FMK). In such cultures the accumulation of cells with a subG1 content (Figure 4D) or Annexin V+ cells (Figure 4E) was markedly reduced. Taken together these results indicate that a caspase 3 dependent pathway is activated in Chk1 depleted cells exposed to thymidine. In contrast to the response of such cells to IR, caspase 3 activation is not dependent upon p53 status although a low level of caspase 2 cleavage can be detected in the p53 deficient cells.

### Response of NBS1-Deficient Cells to Replication Inhibitors

**Figure 5:**
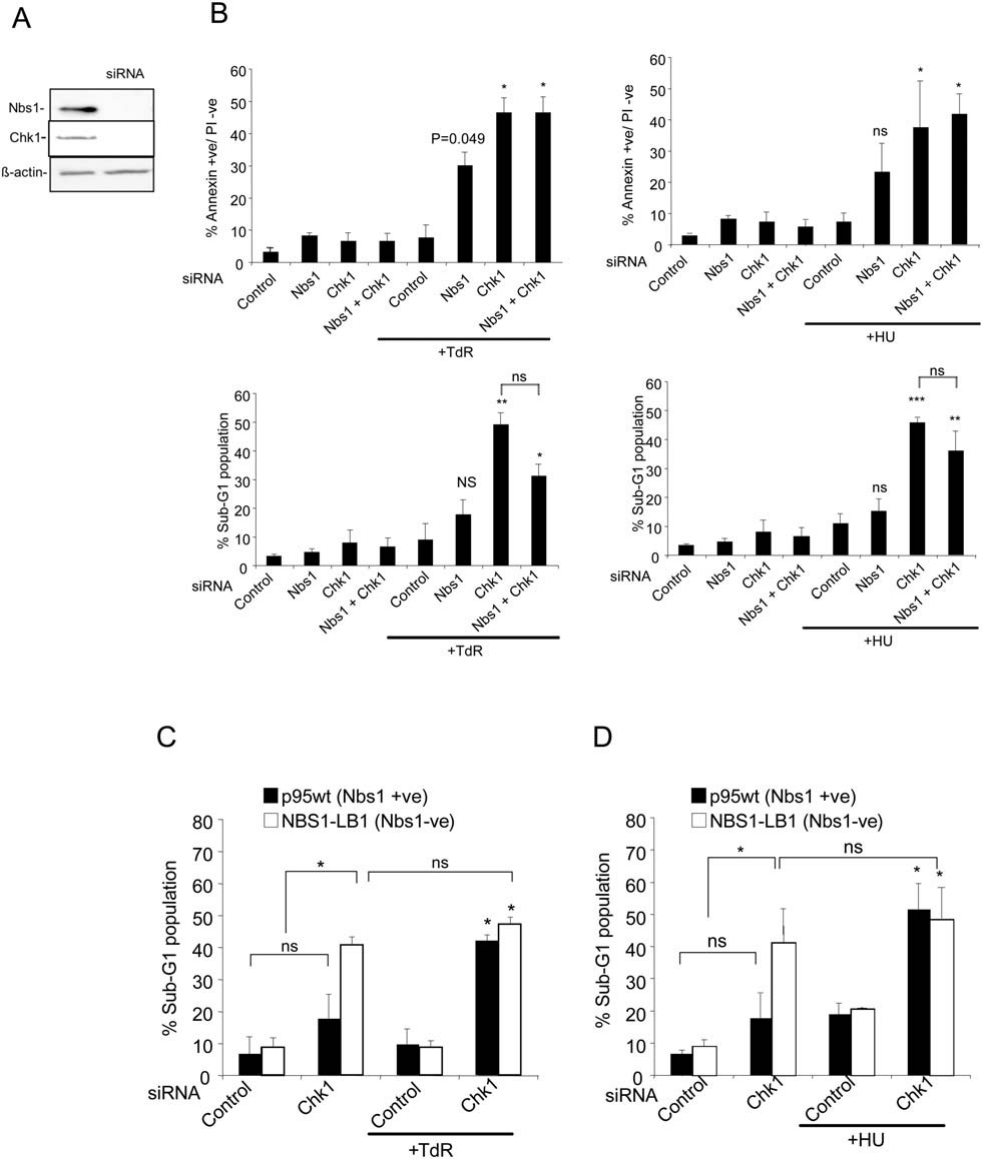
Effect of Nbs1 deficiency on apoptosis in response to replication inhibitors. A) Nbs1 and Chk1 protein levels in HCT116 cells after 48 hours treatment with the siRNAs for the two proteins. B) Induction of apoptosis measured by Annexin V staining (top panels) or sub-G1 population (bottom panels) in HCT116 cells transfected with control, Nbs1 or Chk1 siRNAs singly or in combination exposed or not exposed to 2 mM thymidine or 0.5 mM hyroxyurea. C & D) Induction of apoptosis (measured by the percentage of cells with a sub-G1 DNA content) in Nbs1-deficient (LB1) and corrected (p95wt) fibroblasts transfected with control or Chk1 siRNA following exposure to 2 mM thymidine (C) or 0.5 mM hyroxyurea (D). Results in B–D represent the means of two to three independent experiments±standard deviations. Statistical significance versus thymidine or HU treated control siRNA transfected cells: ns, not significant; *p<.05; **p<.005; ***p<.005.

Recent work has shown that mice carrying a carboxyl terminus deletion of NBS1 are defective in apoptosis in many tissues and in response to IR [9]. To investigate the contribution of NBS1 to apoptosis in response to DNA replication stress, we determined the apoptotic response of HCT116 cultures depleted of NBS1 or immortalized human fibroblasts obtained from Nijmegen breakage syndrome patients to DNA replication inhibitors. HCT116 cultures depleted of NBS1 (Figure 5A) and treated with thymidine showed a slightly reduced level of cells in S- and G2-phases relative to cultures treated with the control siRNA (Figure S3). The fraction of apoptotic cells in thymidine treated cultures reach significance (p = 0.049) when measured by the Annexin V assay, but not in the assay of subG1 cells (Figure 5B). The response of NBS1 depleted cells in either assay was not as robust as that seen with Chk1 depleted cells. Co-depletion of NBS1 and Chk1 did not produce any significant changes in the level of apoptotic cells relative to cells depleted of Chk1 alone. When HCT116 cells depleted of NBS1 were treated with HU, the level of S and G2-phase cells was not greatly affected (Figure S3) and the increase in Annexin V+ or subG1 cells did not reach significance (Figure 5B). The level of Annexin V+ cells in HCT116 cultures depleted of both NBS1 and Chk1 was similar to that of cultures depleted of Chk1 alone.

NBS1−/− fibroblasts (NBS1-LB1) obtained from Nijmegen breakage syndrome patients and fibroblasts corrected for the defect (p95wt, [4] were next examined for their response to replication inhibitors. Thymidine had no significant effect on the level of cells with a subG1 DNA content in either mutant or corrected fibroblast lines treated with the control siRNA while HU produced a small increase in both cell types (Figure 5C & D). Chk1 depletion of the corrected fibroblasts resulted in a ∼two-fold increase in cells with a subG1 DNA content. Intriguingly there was a ∼five-fold increase in the fraction of subG1 cells in Chk1 depleted NBS1−/− fibroblasts (Figure 5C & D). The level of cells with a subG1 DNA content was significantly increased in mutant and corrected fibroblasts treated with Chk1 siRNA relative to cells treated with the control after exposure to either thymidine or HU. However there were no significant differences in the response of the NBS1−/− fibroblasts relative to the corrected cells in these conditions (Figure 5C & D). Interestingly the replication inhibitors did not further increase the level of apoptosis in the NBS1 LB1 (−/−) cells depleted of Chk1.

### Effect of BID Depletion on the Induction of Apoptosis Following Disruption of DNA Replication

**Figure 6:**
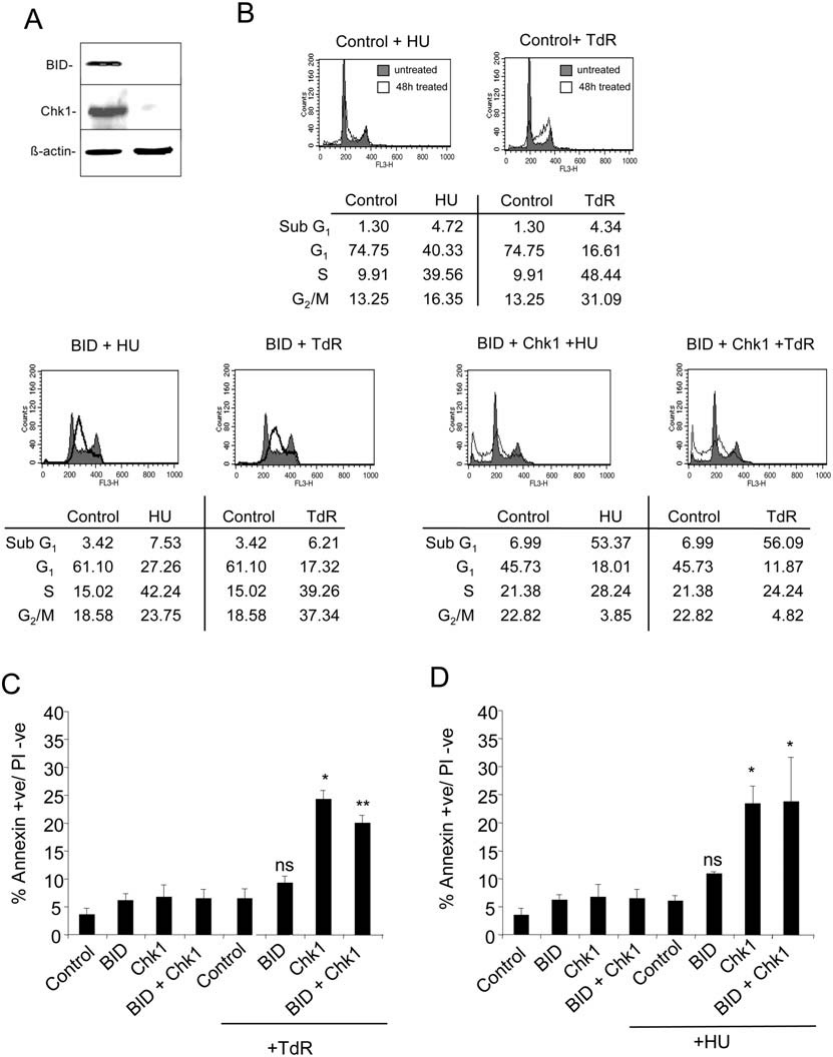
Effect of BID depletion on the induction of apoptosis following disruption of replication. A) Western blot analysis of Chk1 and BID in HCT116 cells treated with control, Chk1 and/or BID siRNAs for 48 hours. β-actin levels are presented as loading controls. B) Representative cell cycle distributions of HCT116 cells transfected with the indicated siRNAs followed by treatment with 2 mM thymidine or 0.5 mM hydroxyurea for 48 hours. Shaded profiles represent control cultures not treated with replication inhibitors, unshaded profiles represent cultures treated with HU or thymidine (TdR). C & D) Induction of apoptosis (measured by Annexin V binding) in HCT116 cells following transfection of the indicated siRNAs exposed or not exposed to 2 mM thymidine (C) or 0.5 mM HU (D) for 48 hours. Results in C & D represent the means of two to three independent experiments±standard deviations. Statistical significance versus thymidine or HU treated control siRNA transfected cells: ns, not significant; *p<.05; **p<.005.

Given the controversy arising over recent reports of an anti-apoptotic role for BID in response to some forms of DNA damage [26],[27], we determined the effect of BID depletion on the induction of apoptosis following treatment with thymdine or HU. Depletion of BID in HCT116 (Figure 6A) had only minor effects on cell cycle distribution after treatment with thymidine or HU (Figure 6B), although, like ATM depleted cells, an accumulation of cells in mid S-phase was detected. Similarly there were only small changes in the level of cells with a subG1 DNA content or AnnexinV+ after treatment of BID depleted cells relative to cells treated with the control siRNA (Figure 6B–D). The strong induction of subG1 or AnnexinV+ cells in cultures depleted of Chk1 after thymidine or HU treatment was not altered in cells depleted of both Chk1 and BID. In addition co-depletion of BID and ATR or BID and ATM had no further effect on the level of apoptotic cells relative to cells treated with ATR or ATM siRNAs alone (Figure S4). Thus BID does not appear to play a major role in the commitment to apoptosis during replication stress in the tumour cells tested here.

## Discussion

In the work reported here we analysed the roles of key proteins controlling the cellular response to DNA damage in the control of apoptosis following DNA replication stress. The role of ATM in promoting apoptosis in response to ionizing radiation is well established [8],[28]. Our data suggest that it plays little or no role in tumour cell lines in response to DNA replication fork stress relative to the ATR-Chk1 pathway. SiRNA mediated depletion of ATM and downstream ATM phosphorylation targets NBS1 and BID had little or no significant effect on the level of apoptotic cells in response to the replication inhibitors in the tumour cell lines tested. We previously reported that Chk2 depletion did not affect the level of apoptotic cells induced by replication inhibitors [17]. In addition early events occurring after the disruption of DNA replication (accumulation of RPA foci and RPA34 hyperphosphorylation) in ATR- or Chk1-depleted cells committed to apoptosis are not detected in ATM-depleted cells. In immortalized fibroblast lines derived from patients with inherited defects in ATM or NBS1, there was no difference in the apoptotic response of mutant cells to replication inhibitors relative to the corrected lines while depletion of Chk1 or ATR gave robust apoptotic responses in all cell types. The only exception to this pattern was NBS1−/− cells that showed an elevated level of apoptosis following Chk1 depletion in the absence of the replication inhibitor. Since thymidine or HU treatment of these cells did not further increase the level of apoptosis, we speculate that the synthetic lethality observed in these conditions may be a consequence of some disruption of replication in these cells.

Depletion of ATR or Chk1 leads to a consistent robust apoptotic response to replication inhibitors in both tumour and immortalized fibroblast cell lines. The response of the ATR-Chk1 pathway is largely directed at the stabilization of DNA replication following stress [29] and it does not appear to be required to activate downstream proapoptotic proteins. The precise event that initiates the death response in the absence of this signalling pathway is not yet clear. Previous work showing that the replication helicase cofactor Cdc45 is required for the Chk1 suppressed apoptotic response suggests that the role of this signalling pathway in maintaining replication fork integrity and preventing firing of new origins following the disruption of DNA replication is critical [20]. Co-depletion of proteins involved in ATR and ATM signalling pathways does not enhance or inhibit the apoptotic response to replication inhibitors indicating that the ATM signalling pathway is not required for the death response. Interestingly, ATR- and ATM-mediated signalling cascades overlap in response to many forms of DNA damage. For example, both pathways stimulate Cdc25A degradation following activation by DNA damage through the action of the Chk1 and Chk2 checkpoint kinases [2],[30]. Thus activation of either pathway can produce S-phase arrest that, in turn, should favour the anti-apoptotic repair of damaged DNA. However, in the absence of Chk1 or ATR, the ATM-mediated response does not appear to be sufficient to protect cells from apoptosis.

It has been reported that the pro-apoptotic protein BID participates in ATM-mediated protein kinase cascade to regulate entry into S-phase and prevent death in response to DNA replication inhibitors. BID showed nuclear localization and was phosphorylated in an ATM-dependent manner following DNA damage in myeloid progenitor cells derived from wild type mice [22]. Furthermore, cells from BID−/− mice showed a strong apoptotic response following treatment that was not evident in BID+/+ cells. In another report mouse embryo fibroblasts obtained from BID−/− mice showed a delayed entry into S-phase but not cell death following exposure to DNA damaging agents [31]. More recently these observations were disputed as several cell types from BID−/− mice generated on a different genetic background failed to show any significant change in S-phase arrest, survival, or apoptosis relative to BID+/+ cells [32]. In our knockdowns of BID in the human colon cancer cell line HCT116, no significant increase in the frequency of apoptotic cells was observed. However, BID depleted cells treated with thymidine accumulated in mid S-phase, suggesting that transition through S-phase was delayed relative to cells treated with the control siRNA.

**Figure 7:**
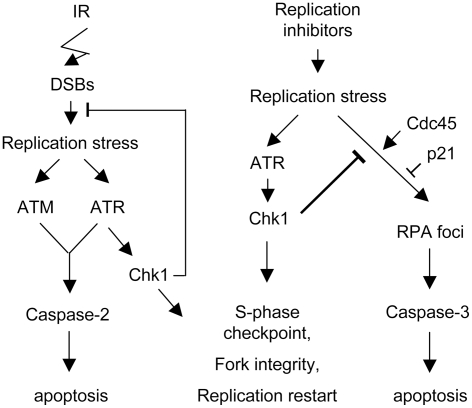
Model of apoptotic pathways suppressed by Chk1 following IR or DNA replication stress. Replication stress developing as a result of DSB formation or processing triggers the ATR/Chk1 protein kinase cascade that suppresses an ATM- and ATR-dependent cleavage of caspase 2 and cell death (left, from [19]). Replication stress triggered by DNA replication inhibitors also triggers the ATR/Chk1 protein kinase cascade that suppresses an apoptotic pathway (right). In Chk1 depleted cells treated with replication inhibitors RPA foci accumulate, caspase 3 is activated, and apoptosis follows. Once this apoptotic response is triggered in the absence of Chk1 or ATR, it does not require ATM or ATR function, unlike the response to IR. The formation of RPA foci and apoptosis are dependent upon Cdc45 function and are modulated by p21 [17],[20].

The data reported here show that the apoptotic pathway suppressed by Chk1 in response to replication inhibitors is clearly distinguishable from both the classical intrinsic death pathway and the Chk1-suppressed IR death response (Figure 7). Unlike the intrinsic pathway, the Chk1 suppressed response to replication inhibitors does not require p53 or Chk2. The Chk1 suppressed death pathway responding to IR is not triggered following depletion of ATR and it requires ATM, ATR and caspase 2 [19]. Although this pathway was identified in screen of p53 deficient zebrafish, p53 deficient human tumour cells treated with Chk1 inhibitors also show caspase 2 cleavage and caspase 2 dependent apoptosis in response to IR. In p53 proficient tumour cells, the cleaved caspase 2 is not detected but caspase 3 is activated under these conditions. In contrast both ATR and Chk1 depleted cells undergo apoptosis in response to replication inhibitors regardless of p53 status and ATM is not required for death. Caspase 3 is clearly activated in both p53 proficient and deficient cell lines. Cleaved caspase 2 is not detected in p53 proficient HCT116 cells and is only weakly induced in the p53 deficient cells. Thus while Chk1 is required for the suppression of apoptosis in response to DNA structural alterations induced by IR or replication stress, the pathways suppressed are distinctly different.

Given the lethal effects of loss of Chk1 function on tumour cells exposed to DNA replication inhibitors, there has been interest in the use of Chk1 inhibitors in chemotherapy. Chk1 is highly expressed in some types of tumours [33]. This may confer some growth advantage to tumour cells as a result of the role of this protein in protecting cells from replication stress that may be induced by hypoxia or nutrient deprivation during tumour development [34]. However loss of Chk1 can also be lethal to some normal cell types [35] and Chk1 knockout mice show embryonic lethality [36]. Nevertheless recent work has shown that Chk1 inhibitors can be used to increase the sensitivity of tumour cells to replication inhibitors *in vitro* and *in vivo* [37]. Furthermore, Chk1 knockdown experiments suggest enhanced lethality for tumour cells may be obtained where the protein is only partially depleted, thus reducing potential lethality caused by complete loss of Chk1. Notably Chk1 inhibitors have been developed that appear to enhance the toxicity of DNA damaging agents in p53 deficient tumour cells but not p53-proficient cells [38] offering prospects for the targeted activation of the Chk1-suppressed apoptotic pathway in at least some types of tumour cells.

## Materials and Methods

### Cell Lines and Cultures

The HCT116 and SW480 human colon cancer cell lines were obtained from American Type Culture Collection (Manassas, VA) while the AT patient derived AT5 cells (TAT5BIVA) was obtained from the European Cell and Culture Collection. HCT116 p53−/− cells were provided by Dr. Bert Vogelstein (Johns Hopkins University, Baltimore, MD). AT-deficient (AT22IJE-T referred to as pEBs here) and corrected (YZ5) cell lines were kindly provided by Dr. Yosef Shiloh (Tel Aviv University, Tel Aviv, Israel). Nbs1 deficient (NBS1-LB1) and corrected (p95wt) fibroblasts were generously provided by Dr. Mike Kastan (St. Jude Children's Research Hospital, Memphis, TN). Cells were maintained in DMEM supplemented with 10% fetal bovine serum (FBS). For experiments using thymidine, dialyzed FBS was used to remove deoxynucleosides in the serum that might interfere in the response to this agent.

### SiRNA Transfection

All siRNAs were obtained from Dharmacon (Lafayette, CO). The ATM and ATR siRNA consisted of a pool of four sequences designed to the relevant DNA sequence. Chk1 siRNAs were designed by J. Blackburn and C. Smythe, (unpublished data). Nbs1 siRNAs (GUCGAUCAGCCGAAAUCAU, CUCACCUUGUCAUGGUAUC, and GCUAGGUUGAUAACAGAAG) were designed by A. Ganesh and the control siRNA was obtained from Eurogentec (OR-0030-NEG). SiRNA duplexes were transfected into cells using Lipofectamine 2000 (Invitrogen, Paisley, United Kingdom) according to manufacturer's instructions. The cells were then incubated for twenty-four hours before further treatment.

### Cell Cycle Analysis

After treatment, floating (obtained from the medium and a PBS wash) and adherent (obtained after trypsinization) cells were pelleted together by centrifugation. Cell pellets were washed with PBS, fixed in 70% ice-cold ethanol, and stored at −20°C for up 2 weeks. Cells were incubated overnight with Propidium Iodide as described previously [17]. Stained nuclei were analyzed on a FACScan (BD Biosciences, Franklin Lakes, NJ) using CellQuest software.

### Detection of Apoptosis

Apoptotic cells were examined using fluorescein isothiocyanate (FITC)-Annexin V and PI detection kit according to the manufacturer's instructions (BD Biosciences). The cells were analyzed by flow cytometry and the percentage of early apoptotic (% Annexin V/PI) cells is presented.

### Immunofluorescence Analysis

Cells were grown on glass coverslips, treated as indicated, fixed with 3% buffered paraformaldehyde for 15 minutes at room temperature (RT) and permeabilized in PBS containing 0.5% Triton X-100 for 8 minutes at RT. Cells were then incubated with 1/250 diluted anti-RPA34 (NA19L; Calbiochem) for 45 minutes at RT and 1∶500 diluted Alexa-594 conjugated anti-mouse IgG (A11005; Molecular Probes, Invitrogen) for 30 minutes at RT and in the dark. Antibody dilutions and washes after incubations were performed in PBS containing 0.5%BSA and 0.05%Tween 20. Coverslips were finally mounted in Vectashield mounting medium with DAPI (H-1500; Vector Laboratories Inc.). For fluorescent analysis, a Nikon Eclipse T200 microscope equipped with a Hamamatsu Orca ER camera and the Volocity 3.6.1 (Improvision) software was used.

### Western Blotting

Cell extracts were prepared and fractionated on SDS-PAGE gels before being blotted onto nitrocellulose (Whatman Schleicher and Schuell, Dassel, Germany) as described previously [21]. Proteins were detected with the ECL detection system (GE Healthcare, Little Chalfont, Buckinghamshire, United Kingdom) using antibodies recognizing ATM (GeneTex Inc, San Antonio, Texas), ATR, (Santa Cruz Biotechnology, Santa Cruz, CA), BID (Santa Cruz Biotechnology) Chk1 (Cell Signaling Technology, Beverly, MA), NBS1 (Cell Signaling Technology), and β-actin (Sigma-Aldrich), RPA34 (NA19L; Calbiochem), Caspase-2 (MAB3507; Millipore), cleaved caspase-3 (ab32042; Abcam).

## Supporting Information

Figure S1 Induction of apoptosis is only weakly increased in HCT116 cells treated with the ATM inhibitor KU-55933 following thymidine treatment. HCT116 cells were treated with control or Chk1 siRNAs and/or 10 µM of the ATM inhibitor KU-55933 before a 48 hour treatment with 2 mM thymidine. Cells were then harvested and the cell cycle distribution of PI stained cells was analysed by flow cytometry. Cells with a subG1 DNA content were scored as apoptotic. Shaded profiles represent control cultures not treated with replication inhibitors, unshaded profiles represent cultures treated with thymidine (TdR).
(0.92 MB TIF)

Figure S2 Effects of ATM, ATR or Chk1 depletion on the induction of apoptosis following replication fork stress in SW480 cells. Induction of apoptosis in ATM-, ATR, or Chk1-depleted SW480 cells measured by the percentage of population having a sub-G1 DNA content following 48 hour treatment with 0.5 mM HU. Results represent the means of two to three independent experiments±standard deviations. Statistical significance versus HU treated control siRNA transfected cells: ns, not significant; *p<.05.
(0.42 MB TIF)

Figure S3 Cell cycle analysis of HCT116 cells treated with Chk1 and NBS1 siRNAs singly or in combination in the presence or absence of thymidine. HCT116 cells were transfected with the indicated siRNAs and after 48 hours were treated with 2 mM thymidine or 0.5 mM HU for 48 hours. Cells were then PI stained and analysed for DNA content by flow cytometry. Shaded profiles represent control cultures not treated with replication inhibitors, unshaded profiles represent cultures treated with HU or thymidine (TdR).
(0.88 MB TIF)

Figure S4 BID depletion has no significant effect on the induction of apoptosis following disruption of DNA replication. Induction of apoptosis in HCT116 cells transfected with the indicated siRNAs (A) singly or in combination and treated or not treated with 2 mM thymidine or 0.5 mM hydroxyurea. Apoptosis was measured by the percentage of cells with a subG1 DNA content (B) or those that were Annexin V+/PI negative (C & D). Results represent the means of two to three independent experiments±standard deviations. Statistical significance versus HU treated control siRNA transfected cells: ns, not significant; *p<.05; **p<.005.
(0.81 MB TIF)
