# Supplementary figures and images for: ATR and Chk1 Suppress a Caspase-3–Dependent Apoptotic Response Following DNA Replication Stress
Source: PLoS Genet. 2009 Jan 2;5(1):e1000324. doi: 10.1371/journal.pgen.1000324 (PMC2607051; doi:10.1371/journal.pgen.1000324)

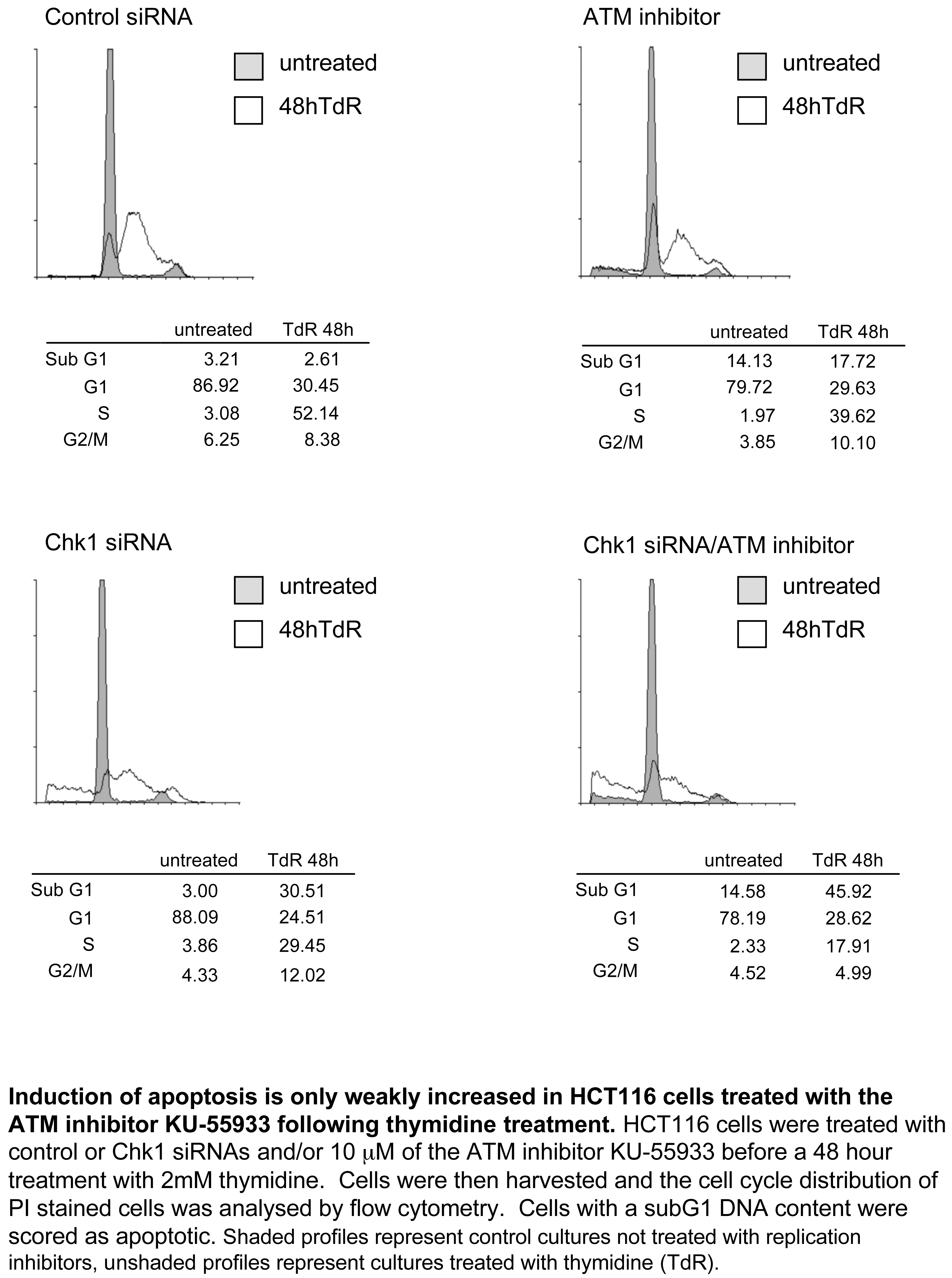

Supplement: Figure S1 — Induction of apoptosis is only weakly increased in HCT116 cells treated with the ATM inhibitor KU-55933 following thymidine treatment. HCT116 cells were treated with control or Chk1 siRNAs and/or 10 µM of the ATM inhibitor KU-55933 before a 48 hour treatment with 2 mM thymidine. Cells were then harvested and the cell cycle distribution of PI stained cells was analysed by flow cytometry. Cells with a subG1 DNA content were scored as apoptotic. Shaded profiles represent control cultures not treated with replication inhibitors, unshaded profiles represent cultures treated with thymidine (TdR). (0.92 MB TIF) [file pgen.1000324.s001.tif]

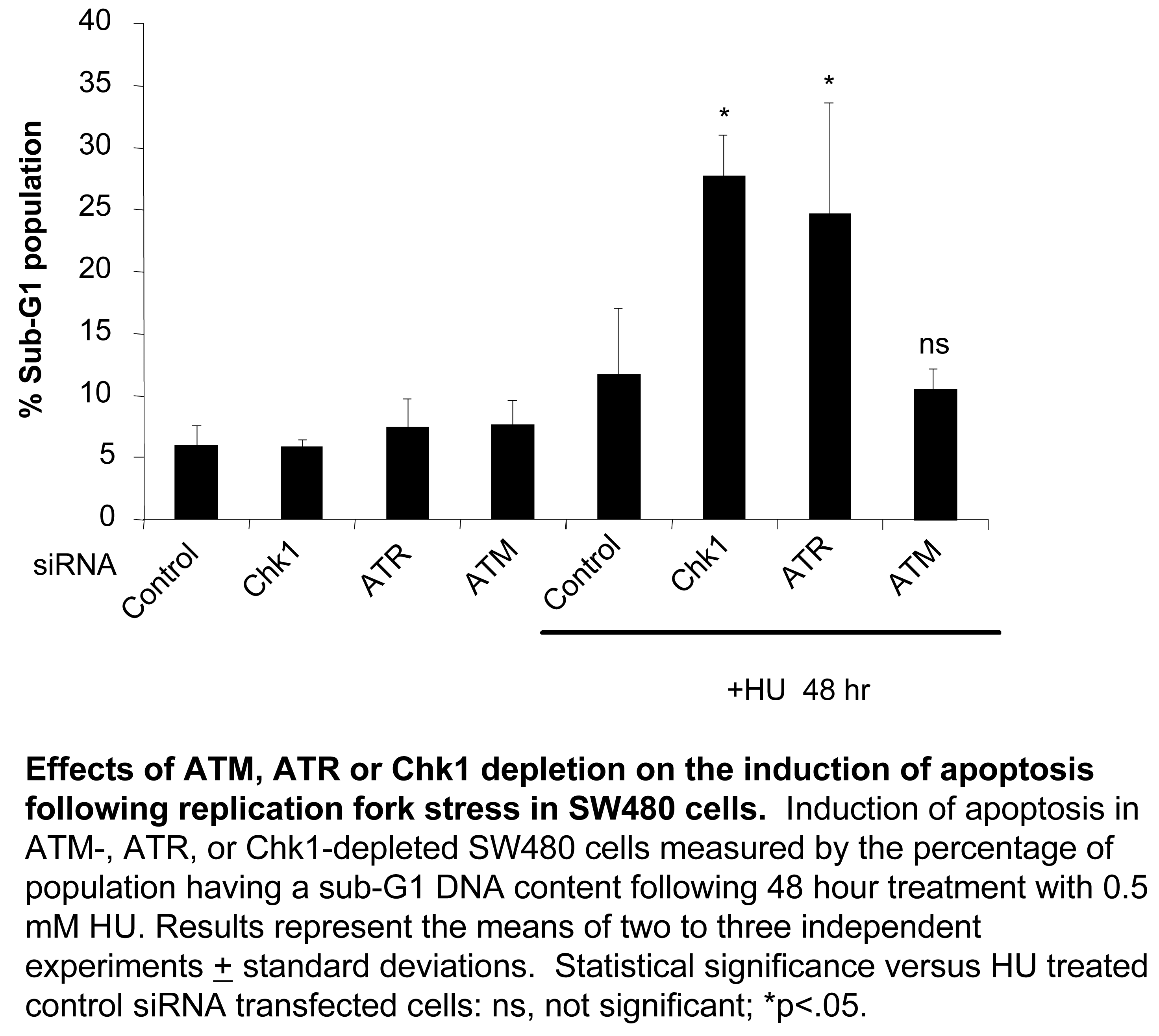

Supplement: Figure S2 — Effects of ATM, ATR or Chk1 depletion on the induction of apoptosis following replication fork stress in SW480 cells. Induction of apoptosis in ATM-, ATR, or Chk1-depleted SW480 cells measured by the percentage of population having a sub-G1 DNA content following 48 hour treatment with 0.5 mM HU. Results represent the means of two to three independent experiments±standard deviations. Statistical significance versus HU treated control siRNA transfected cells: ns, not significant; *p<.05. (0.42 MB TIF) [file pgen.1000324.s002.tif]

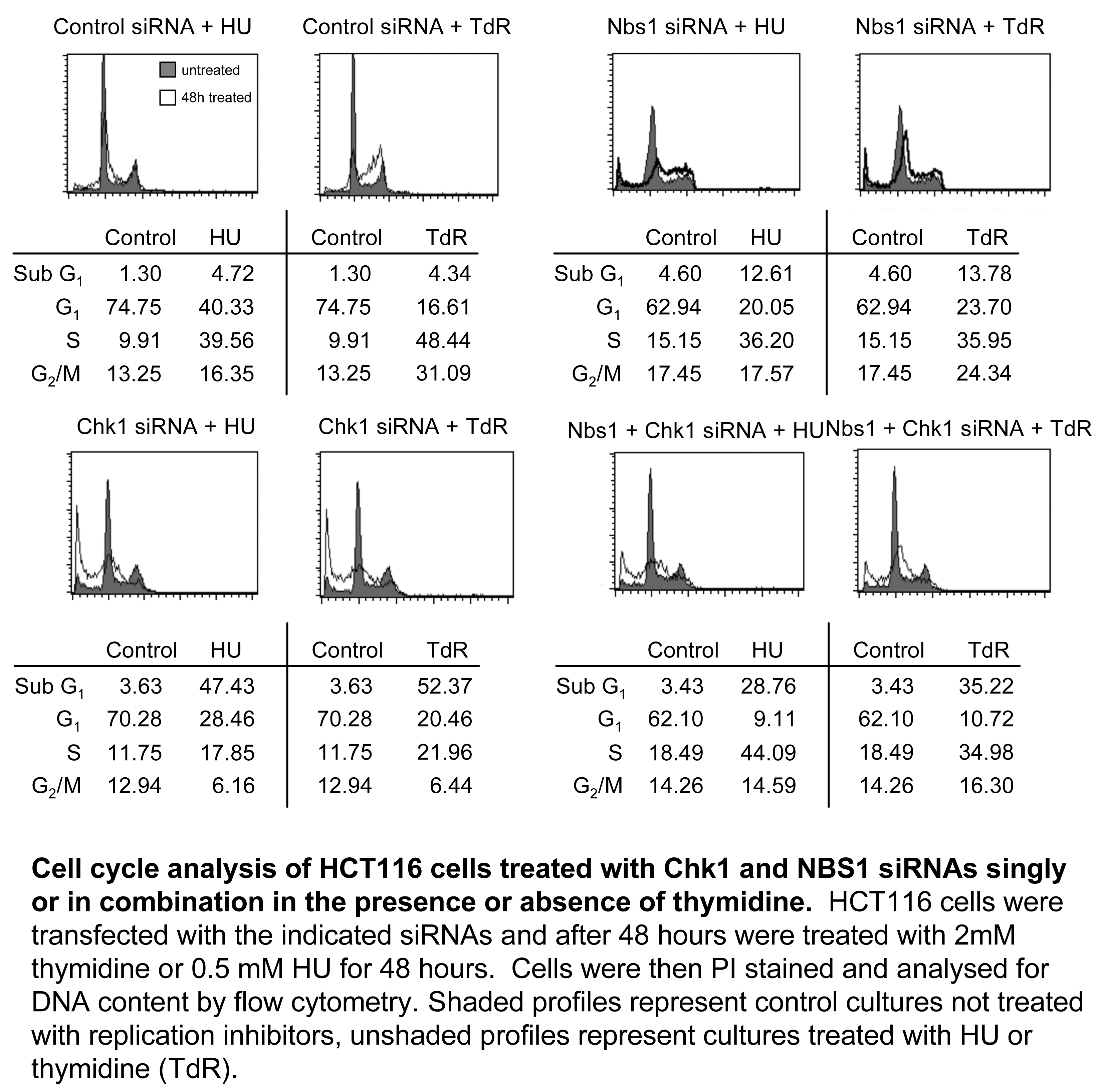

Supplement: Figure S3 — Cell cycle analysis of HCT116 cells treated with Chk1 and NBS1 siRNAs singly or in combination in the presence or absence of thymidine. HCT116 cells were transfected with the indicated siRNAs and after 48 hours were treated with 2 mM thymidine or 0.5 mM HU for 48 hours. Cells were then PI stained and analysed for DNA content by flow cytometry. Shaded profiles represent control cultures not treated with replication inhibitors, unshaded profiles represent cultures treated with HU or thymidine (TdR). (0.88 MB TIF) [file pgen.1000324.s003.tif]

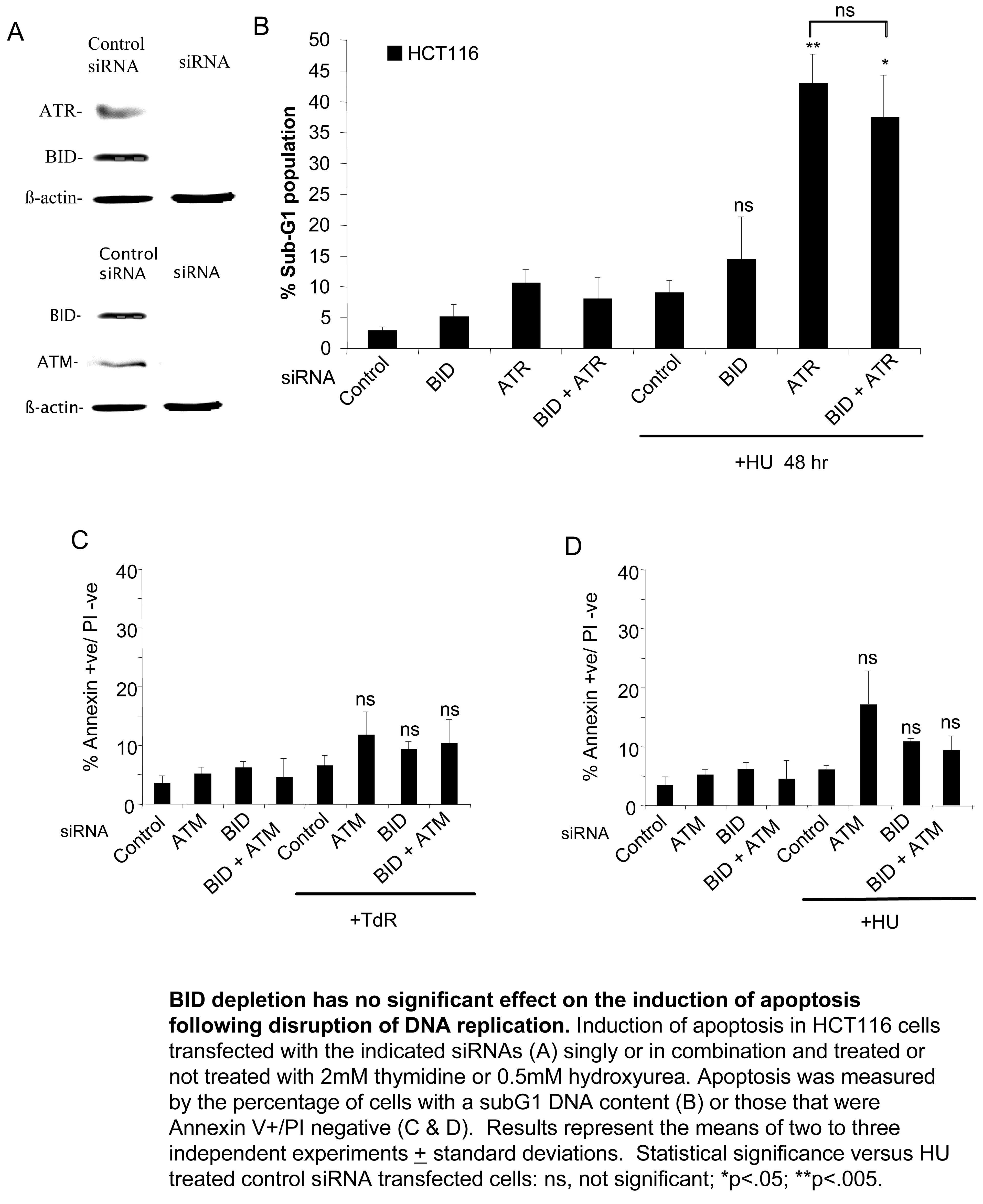

Supplement: Figure S4 — BID depletion has no significant effect on the induction of apoptosis following disruption of DNA replication. Induction of apoptosis in HCT116 cells transfected with the indicated siRNAs (A) singly or in combination and treated or not treated with 2 mM thymidine or 0.5 mM hydroxyurea. Apoptosis was measured by the percentage of cells with a subG1 DNA content (B) or those that were Annexin V+/PI negative (C & D). Results represent the means of two to three independent experiments±standard deviations. Statistical significance versus HU treated control siRNA transfected cells: ns, not significant; *p<.05; **p<.005. (0.81 MB TIF) [file pgen.1000324.s004.tif]
